# Supplementary material for: Pharmacodynamics of Five Anthraquinones (Aloe-emodin, Emodin, Rhein, Chysophanol, and Physcion) and Reciprocal Pharmacokinetic Interaction in Rats with Cerebral Ischemia
Source: Molecules. 2019 May 17;24(10):1898. doi: 10.3390/molecules24101898 (PMC6571683; doi:10.3390/molecules24101898)
Supplement: Supplementary file 1 [file molecules-24-01898-s001.pdf]

# Supplementary Materials

Matrix effect and extraction recovery experiments:

According to the preparation method of mixed reference materials, the mixed reference materials with high, medium and low concentrations were prepared respectively. Then the peak areas (A1) of the five aglycones and internal standard were determined respectively according to "4.3.1 Apparatus and operation conditions". Blank plasma samples were spiked with mixed reference at the above high, medium and low concentrations and treated as the “4.3.2 Plasma sample preparation”. Then the peak areas (A2) of the five aglycones and internal standard were determined respectively. The quality control samples with high, medium and low concentrations were directly determined according to the sample treatment method, Then the peak areas (A3) of the five aglycones and internal standard were determined respectively. Each concentration was measured in parallel for 6 times. The matrix effect is the ratio of the peak area (A2) to the peak area (A1) of the corresponding component. The extraction recovery ratio is the ratio of the peak area (A3) to the peak area (A2) of the corresponding components.

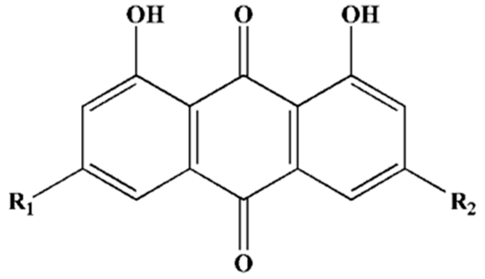


| | $R_1$ | $R_2$ |
|---|---|---|
| **Rhein** | H | COOH |
| **Emodin** | OH | $CH_3$ |
| **Chrysophanol** | H | $CH_3$ |
| **Aloe-emodin** | H | $CH_2OH$ |
| **Physcion** | $OCH_3$ | $CH_3$ |
| **1,8-Dihydroxyanthraquinone** | -- | -- |

**Figure S1.** Chemical formula for five anthraquinones (Rhein, Emodin, Chrysophanol, Aloe-emodin, Physcion) and IS.

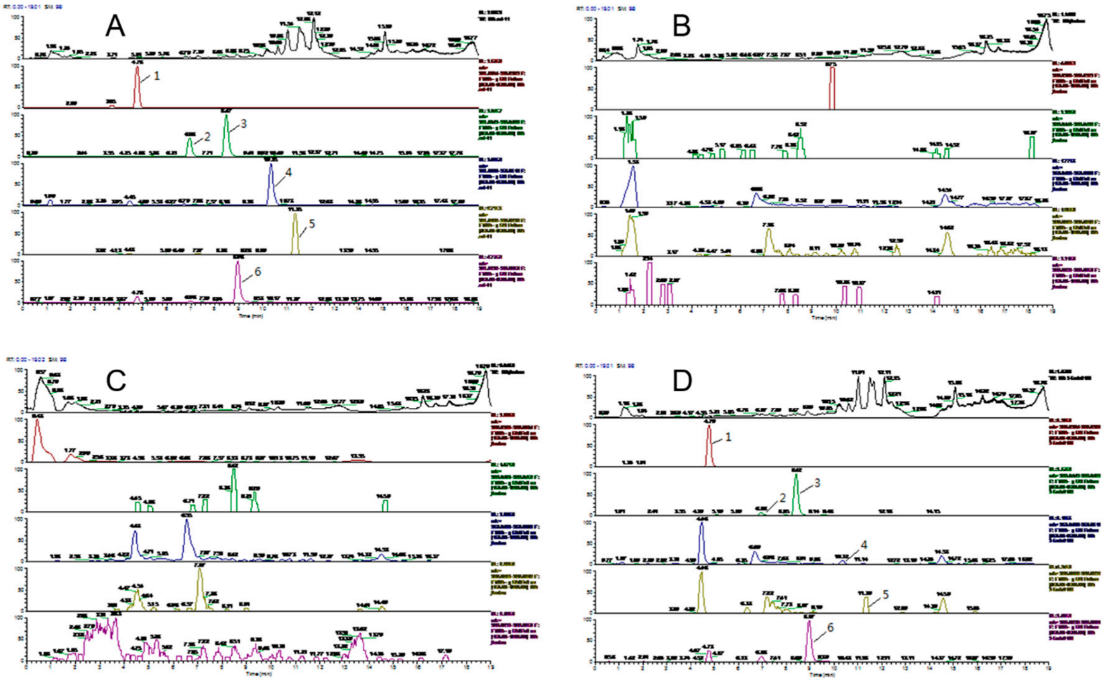


**Figure S2.** Six-channel chromatogram of five anthraquinones (Aloe-emodin, Aloe, Rhein, Chrysophanol, Pphyscion). A: Mixed reference. B: Blank methanol. C: Blank plasma sample. D: Drug-containing plasma sample.

**Table S1.** Calibration curves for the 5 anthraquinones in rat plasma

| Analytes | Regression equation | r | Linear range (ng/mL) | LLOQ (ng/mL) | LOQ (ng/mL) |
|---|---|---|---|---|---|
| Aloe-emodin | $Y = 1.666{\times}10^{-2}x + 7.236{\times}10^{-3}$ | 0.9987 | 0.68-3424 | 0.68 | 0.026 |
| Emodin | $Y = 2.318{\times}10^{-1}x - 1.214{\times}10^{-3}$ | 0.9971 | 0.74-3728 | 0.74 | 0.098 |
| Rhein | $Y = 8.005{\times}10^{-4}x + 1.561{\times}10^{-3}$ | 0.9997 | 0.66-3312 | 0.66 | 0.098 |
| Chrysophanol | $Y = 2.896{\times}10^{-2}x - 7.059{\times}10^{-3}$ | 0.9989 | 0.62-3104 | 0.62 | 0.538 |
| Physcion | $Y=1.437{\times}10^{-1}x + 1.340{\times}10^{-1}$ | 0.9996 | 0.69-3456 | 0.69 | 0.317 |

**Table S2.** Intra-day and inter-day precision and accuracy for determination of the 5 anthraquinones in rat plasma (mean ± SD, n = 6, 5 replicates per day for 5 days)

| Analytes | Spiked concentration (ng/mL) | Intra-day | | | Inter-day | | |
|---|---|---|---|---|---|---|---|
| | | Concentration (ng/mL) | Accuracy (%) | RSD (%) | Concentration (ng/mL) | Accuracy (%) | RSD (%) |
| Aloe-emodin | 9.32 | 8.99 ± 0.55 | -3.54 | 6.12 | 8.95 ± 0.59 | -3.97 | 6.59 |
| | 93.2 | 90.19 ± 7.07 | -3.23 | 7.84 | 87.03 ± 5.18 | -6.62 | 5.95 |
| | 932 | 926.5 ± 10.53 | -0.59 | 1.14 | 915.7 ± 8.6 | -1.75 | 0.94 |
| Emodin | 7.76 | 6.91 ± 0.5 | -10.95 | 7.24 | 6.79 ± 0.26 | -12.50 | 3.83 |
| | 77.6 | 73.23 ± 1.48 | -5.63 | 2.02 | 71.61 ± 3.55 | -7.72 | 4.96 |
| | 776 | 784.2 ± 8.21 | 1.06 | 1.05 | 773.5 ± 13.34 | -0.32 | 1.72 |
| Rhein | 8.28 | 7.77 ± 0.67 | -6.16 | 8.62 | 6.75 ± 0.01 | -18.48 | 0.15 |
| | 82.8 | 63.89 ± 4.24 | -22.84 | 6.64 | 65.86 ± 3.19 | -20.46 | 4.84 |
| | 828 | 792.2 ± 37.18 | -4.32 | 4.69 | 796.2 ± 35.13 | -3.84 | 4.41 |
| Chrysophanol | 8.56 | 7.65 ± 0.41 | -10.63 | 5.36 | 7.72 ± 0.31 | -9.81 | 4.02 |
| | 85.6 | 79.75 ± 4.39 | -6.83 | 5.50 | 79.75 ± 4.39 | -6.83 | 5.50 |
| | 856 | 856.9 ± 14.17 | 0.11 | 1.65 | 844.9 ± 11.91 | -1.30 | 1.41 |
| Physcion | 8.64 | 7.09 ± 0.31 | -17.94 | 4.37 | 7.08 ± 0.31 | -18.06 | 4.38 |
| | 86.4 | 77.25 ± 3.76 | -10.59 | 4.87 | 78.86 ± 6.17 | -8.73 | 7.82 |
| | 864 | 881.4 ± 4.18 | 2.01 | 0.47 | 877.2 ± 9.06 | 1.53 | 1.03 |

**Table S3.** Matrix effects and extraction recovery of the 5 anthraquinones in rat plasma (mean ± SD, n = 6).

| Analytes | Spiked concentration(ng/mL) | Matrix effect | | Extraction recovery | |
|---|---|---|---|---|---|
| | | Mean (%) | RSD (%) | Mean (%) | RSD (%) |
| Aloe-emodin | 9.32 | 99.03 | 3.78 | 81.09 | 4.85 |
| | 93.2 | 99.85 | 5.32 | 88.76 | 3.82 |
| | 932 | 104.9 | 4.2 | 102.8 | 1.43 |
| Emodin | 7.76 | 102.8 | 3.98 | 82.17 | 3.2 |
| | 77.6 | 112.4 | 4.21 | 93.76 | 2.78 |
| | 776 | 103.9 | 4.78 | 99.81 | 3.65 |
| Rhein | 8.28 | 99.82 | 2.98 | 75.63 | 3.21 |
| | 82.8 | 100.3 | 3.45 | 83.54 | 5.43 |
| | 828 | 98.78 | 4.67 | 93.78 | 5.32 |
| Chrysophanol | 8.56 | 79.89 | 0.48 | 84.63 | 5.34 |
| | 85.6 | 88.92 | 6.32 | 87.56 | 6.56 |
| | 856 | 76.09 | 4.76 | 98.45 | 5.32 |
| Physcion | 8.64 | 115 | 6.34 | 79.04 | 4.58 |
| | 86.4 | 103.9 | 4.34 | 84.78 | 2.38 |
| | 864 | 110.4 | 4.89 | 101.7 | 3.4 |

**Table S4.** Stability of the 5 anthraquinones in rat plasma (mean ± SD, n = 6)

| Analytes | Spiked concentration (ng/mL) | Short-term stability | | Freeze/thaw stability | | Long-term stability | |
|---|---|---|---|---|---|---|---|
| | | Concentration (ng/mL) | Accuracy (%) | Concentration (ng/mL) | Accuracy (%) | Concentration (ng/mL) | Accuracy (%) |
| Aloe-emodin | 9.32 | 8.88 ± 0.38 | -4.72 | 8.73 ± 0.4 | -6.33 | 8.65 ± 0.3 | -7.19 |
| | 93.2 | 90.75 ± 4.54 | -2.63 | 92.73 ± 2.0 | -0.50 | 92.33 ± 1.35 | -0.93 |
| | 932 | 925.8 ± 8.7 | -0.67 | 925.6 ± 8.67 | -0.69 | 931.2 ± 7.16 | -0.09 |
| | 7.76 | 6.85 ± 0.33 | -11.73 | 7.42 ± 0.46 | -4.38 | 7.26 ± 0.36 | -6.44 |
| Emodin | 77.6 | 74.67 ± 3.12 | -3.78 | 76.0 ± 4.55 | -2.06 | 72.39 ± 2.08 | -6.71 |
| | 776 | 780.4 ± 10.12 | 0.57 | 767.1 ± 12.3 | -1.15 | 766.1 ± 8.6 | -1.28 |
| | 8.28 | 7.84 ± 0.45 | -5.31 | 7.8 ± 0.35 | -5.80 | 7.94 ± 0.28 | -4.11 |
| Rhein | 82.8 | 77.33 ± 4.64 | -6.61 | 80.73 ± 2.39 | -2.50 | 80.24 ± 3.13 | -3.09 |
| | 828 | 807.6 ± 29.1 | -2.46 | 823.4 ± 9.51 | -0.56 | 817.6 ± 15.28 | -1.26 |
| | 8.56 | 7.71 ± 0.35 | -9.93 | 8.32 ± 0.2 | -2.80 | 8.28 ± 0.17 | -3.27 |
| Chrysophanol | 85.6 | 80.45 ± 3.85 | -6.02 | 84.54 ± 2.99 | -1.24 | 83.24 ± 0.73 | -2.76 |
| | 856 | 856.0 ± 11.72 | 0.00 | 856.5 ± 16.36 | 0.06 | 845.9 ± 5.72 | -1.18 |
| | 8.64 | 7.16 ± 0.28 | -17.13 | 7.80 ± 0.23 | -9.72 | 7.42 ± 0.37 | -14.12 |
| Physcion | 86.4 | 77.6 ± 3.15 | -10.19 | 84.64 ± 3.86 | -2.04 | 81.07 ± 4.91 | -6.17 |
| | 864 | 877.4 ± 8.67 | 1.55 | 863.4 ± 8.44 | -0.07 | 872.2 ±5.74 | 0.95 |

I Short-term stability was assessed by analyzing samples kept at 4 ℃ for 24 h.

II Freeze–thaw stability was evaluated at three consecutive freeze–thaw cycles (−20 °C to room temperature as one cycle).

III Long-term stability was studied by assaying samples following a period of 30 days of storage at -80 °C.
